# Supplementary material for: The impact of chest CT body composition parameters on clinical outcomes in COVID-19 patients
Source: PLoS One. 2021 May 14;16(5):e0251768. doi: 10.1371/journal.pone.0251768 (PMC8121324; doi:10.1371/journal.pone.0251768)
Supplement: S1 Table — Spearman correlations between BMI and CT fat distribution parameters. Spearman’s rank correlation coefficients are reported with respective p-values between brackets. CT: Computed Tomography; IMAT: intermuscular adipose tissue area; SAT: subcutaneous adipose tissue area; TAT: total adipose tissue area; VAT: visceral adipose tissue area. (PDF) [file pone.0251768.s001.pdf]

	BMI (n=88)	TAT	VAT	SAT	IMAT
BMI	1				
TAT	0.7059 (0.000)	1			
VAT	0.5053 (0.000)	0.5536 (0.000)	1		
SAT	0.6304 (0.000)	0.9590 (0.000)	0.3613 (0.001)	1	
IMAT	0.6119 (0.000)	0.6934 (0.000)	0.6452 (0.000)	0.5357 (0.000)	1
