# Supplementary material for: The impact of chest CT body composition parameters on clinical outcomes in COVID-19 patients
Source: PLoS One. 2021 May 14;16(5):e0251768. doi: 10.1371/journal.pone.0251768 (PMC8121324; doi:10.1371/journal.pone.0251768)
Supplement: S2 Table — β coefficients with respective 95% confidence intervals are reported. CT: Computed Tomography; IMAT: intermuscular adipose tissue area; L/S: liver to spleen ratio; TAT: total adipose tissue area; VAT: visceral adipose tissue area. (PDF) [file pone.0251768.s002.pdf]

Variables	Hospitalization		Mechanical ventilation or death		Death	
	β	**95% CI**	***β***	**95% CI**	***β***	**95% CI**
**Pectoral area**						
I quart [6-12]	0		0		0	
II quart [12.1-17]	0.288	-0.358;0.933	-0.240	-0.895;0.415	-0.014	-0.804;0.775
III quart [17.1-21]	-0.045	-0.710;0.620	-0.112	-0.798;0.573	0.176	-0.635;0.987
IV quart [21.1-50]	-0.316	-0.957;0.325	-0.370	-1.061;0.321	-0.584	-1.506;0.338
**Pectoral density**						
I quart [3-27]	0		0		0	
II quart [28-34]	-0.591	-1.288;0.105	-0.910	-1.592;-0.229	-0.587	-1.352;0.179
III quart [35-41]	-0.434	-1.135;0.267	-0.551	-1.195;0.094	-0.925	-1.744;-0.107
IV quart [41.1-63]	-1.639	-2.334;-0.945	-1.252	-1.99;-0.514	-1.406	-2.369;-0.443
**L/S ratio**						
I quart [0.15-0.920]	0		0		0	
II quart [0.920-1.059]	-0.199	-0.913;0.516	-0.055	-0.702;0.593	0.808	-0.002;1.619
III quart [1.059-1.76]	-1.033	-1.711;-0.355	-0.767	-1.462;-0.071	-0.029	-0.930;0.871
IV quart [1.76-2.25]	-0.892	-1.581;-0.203	-0.683	-1.382;0.017	0.149	-0.739;1.036
**TAT**						
I quart [20- 159]	1		0		0	
II quart [160-223]	0.620	-0.077;1.316	0.507	-0.245;1.260	0.098	-0.770;0.966
III quart [224-292]	0.488	-0.200;1.177	0.157	-0.620;0.934	0.000	-0.882; 0.882
IV quart [293-649]	0.758	0.051;1.464	0.761	0.019;1.502	0.000	-0.882; 0.882
**VAT**						
I quart [2-23]	0		0		0	
II quart [24-34]	0.831	0.202;1.461	1.619	0.757;2.481	1.517	0.366; 2.668
III quart [35-47]	1.001	0.348;1.653	1.312	0.425;2.199	1.341	0.161;2.520
IV quart [48-118]	1.941	1.187;2.695	2.185	1.329;3.042	2.148	1.031;3.266
**IMAT**						
I quart [0-18]	0		0		0	
II quart [19-27]	1.502	0.791;2.212	1.378	0.481;2.275	1.341	0.259; 2.423
III quart [28-37]	1.523	0.814;2.232	1.491	0.602;2.381	0.913	-0.216;2.041
IV quart [38-83]	2.100	1.318;2.882	2.140	1.263;3.017	1.891	0.847; 2.935
