# Supplementary material for: The impact of chest CT body composition parameters on clinical outcomes in COVID-19 patients
Source: PLoS One. 2021 May 14;16(5):e0251768. doi: 10.1371/journal.pone.0251768 (PMC8121324; doi:10.1371/journal.pone.0251768)
Supplement: S3 Table — Hospitalization, ventilation and/or death, and mortality OR with 95% CI are reported for unit increase of CT body composition parameters (HU for pectoral density and cm2 for VAT and TAT) and for IMAT quartiles. The same model is reported after excluding all patients with comorbidities, only patients with diabetes, only patients with cardiovascular comorbidities, including hypertension, and only patients with previous cancer diagnosis. OR adj: adjusted for age, sex, and calendar period. HU: Hounsfield Unit; IMAT: intermuscular adipose tissue area; TAT: total adipose tissue area; VAT: visceral adipose tissue area. a as continuous variable (for one unit increase). (PDF) [file pone.0251768.s003.pdf]

All patients	Hospitalization		Mechanical ventilation or death		Death	
	OR adj	95% CI	OR adj	95% CI	OR adj	95% CI
Pectoral density[a]	0.967	0.935-1.000	0.964	0.934-0.996	0.962	0.922-1.004
TAT[a]	1.005	1.002-1.008	1.005	1.002-1.009	1.002	0.998-1.007
VAT[a]	1.028	1.008-1.049	1.026	1.008-1.043	1.017	0.997-1.038
IMAT						
I quart [0-18]					1	
II quart [19-27]					1.190	0.308-4.588
III quart [28-37]					0.820	0.203-3.312
IV quart [38-83]					1.615	0.431-6.053
IMAT[a]	1.028	1.006-1.050	1.024	1.005-1.043		

After excluding all patients with comorbidities	Hospitalization		Mechanical Ventilation or death		Death	
	OR adj	95% CI	OR adj	95% CI	OR adj	95% CI
Pectoral density[a]	0.984	0.948-1.021	0.991	.950-1.035	0.998	0.941-1.059
T7-T8 TAT[a]	1.004	1.000-1.008	1.002	0.997-1.006	0.998	0.992-1.005
T7-T8 VAT[a]	1.024	1.002-1.047	1.023	1.001-1.045	1.016	0.988-1.045
IMAT						
I quart [0-18]					1	
II quart [19-27]					1.877	0.333-10.592
III quart [28-37]					0.445	0.062-3.184
IV quart [38-83]					0.982	0.161-5.977
T7-T8 IMAT[a]	1.019	0.996-1.042	1.003	0.979-1.028		

**After excluding patients with diabetes**	**Hospitalization**		**Mechanical Ventilation or death**		**Death**	
	**OR adj**	**95% CI**	**OR adj**	**95% CI**	**OR adj**	**95% CI**
Pectoral density[a]	0.970	0.936-1.006	0.978	0.941-1.017	0.981	0.934-1.032
T7-T8 TAT[a]	1.004	1.001-1.008	1.003	0.999-1.006	1.001	0.996-1.005
T7-T8 VAT[a]	1.026	1.006-1.048	1.016	0.997-1.035	1.007	0.985-1.030
IMAT						
I quart [0-18]					1	
II quart [19-27]					1.657	0.415-6.620
III quart [28-37]					0.948	0.222-4.049
IV quart [38-83]					1.614	0.405-6.439
T7-T8 IMAT[a]	1.024	1.002-1.046	1.013	0.993-1.035		

***After* excluding cardiovascular comorbidities**	**Hospitalization**		**Mechanical ventilation or death**		**Death**	
	**OR adj**	**95% CI**	**OR adj**	**95% CI**	**OR adj**	**95% CI**
Pectoral density[a]	0.985	0.951-1.021	0.981	0.945-1.018	0.980	0.928-1.036
T7-T8 TAT [a]	1.004	1.001-1.008	1.005	1.001-1.009	1.000	0.994-1.006
T7-T8 VAT [a]	1.023	1.001-1.045	1.027	1.006-1.049	1.015	0.988-1.044
IMAT						
I quart [0-18]					1	
II quart [19-27]					2.124	0.328-13.772
III quart [28-37]					0.785	0.108-5.674
IV quart [38-83]					1.552	0.248-9.720
T7-T8 IMAT[a]	1.022	0.999-1.045	1.018	0.996-1.040	1.010	0.981-1.040

After excluding patients with previous cancer diagnosis	**Hospitalization**		**Mechanical Ventilation or death**		**Death**	
	**OR adj**	**95% CI**	**OR adj**	**95% CI**	**OR adj**	**95% CI**
Pectoral density[a]	0.967	0.933-1.003	0.966	0.932-1.001	0.982	0.936-1.031
T7-T8 TAT[a]	1.004	1.001-1.008	1.005	1.002-1.009	1.003	0.998-1.008
T7 T8 VAT[a]	1.026	1.003-1.049	1.031	1.009-1.053	1.018	0.992-1.045
IMAT						
I quart [0-18]					1	
II quart [19-27]					2.180	0.348-13.669
III quart [28-37]					1.049	0.162-6.768
IV quart [38-83]					2.190	0.356-13.480
T7-T8 IMAT[a]	1.027	1.003-1.051	1.025	1.003-1.048		
