# Supplementary material for: The impact of chest CT body composition parameters on clinical outcomes in COVID-19 patients
Source: PLoS One. 2021 May 14;16(5):e0251768. doi: 10.1371/journal.pone.0251768 (PMC8121324; doi:10.1371/journal.pone.0251768)
Supplement: S4 Table — P* Pearson’s chi-squared test and p-value for the hypothesis of independence in the two-way table. IMAT: intermuscular adipose tissue area; TAT: total adipose tissue area; VAT: visceral adipose tissue area. (PDF) [file pone.0251768.s004.pdf]

	Age quartiles						
	Total	[17-53)	[53-66)	[66-76)	[76-96]	P*	Spearman's ρ
**Pectoral density** [I quart: 3-27]	85 (27.3)	7 (8.8)	24 (30.8)	30 (38.0)	24 (32.4)	0.000	-0.308 (0.000)
[II quart: 28-34]	76 (24.4)	14 (17.5)	18 (23.1)	18 (22.8)	26 (35.1)		
[III quart: 35-41]	78 (25.1)	25 (31.3)	17 (21.8)	18 (22.8)	18 (24.3)		
[IV quart: 41.1-63]	72 (23.2)	34 (42.5)	19 (24.4)	13 (16.5)	6 (8.1)		
**TAT** [I quart: 20- 159]	68 (25.0)	25 (37.9)	13 (20.0)	11 (15.9)	19 (26.4)	0.017	0.050 (0.413)
[II quart: 160-234]	68 (25.0)	9 (13.6)	15 (23.1)	19 (27.5)	25 (34.7)		
[III quart: 224-292]	68 (25.0)	18 (27.3)	14 (21.5)	20 (29.0)	16 (22.2)		
[IV quart: 293-649]	68 (25.0)	14 (21.2)	23 (35.4)	19 (27.5)	12 (16.7)		
**VAT** [I quart: 2-23]	83 (26.5)	41 (51.3)	19 (24.4)	11 (14.1)	12 (15.6)	0.000	0.390 (0.000)
[II quart: 24-34]	80 (25.6)	22 (27.5)	20 (25.6)	16 (20.5)	22 (28.6)		
[III quart: 35-47]	74 (23.6)	12 (15.0)	21 (26.9)	26 (33.3)	15 (19.5)		
[IV quart: 48-118]	76 (24.3)	5 (6.3)	18 (23.1)	25 (32.1)	28 (36.4)		
**IMAT** [I quart: 0-18]	76 (27.6)	41 (62.1)	18 (26.9)	9 (13.0)	8 (11.0)	0.000	0.402 (0.000)
[II quart: 19-27]	66 (24.0)	9 (13.6)	16 (23.9)	20 (29.0)	21 (28.8)		
[III quart: 28-37]	67 (24.4)	10 (15.2)	21 (31.3)	18 (26.1)	18 (24.7)		
[IV quart: 38-83]	66 (24.0)	6 (9.1)	12 (17.9)	23 (31.9)	26 (35.6)		
