# Supplementary figures and images for: The impact of chest CT body composition parameters on clinical outcomes in COVID-19 patients
Source: PLoS One. 2021 May 14;16(5):e0251768. doi: 10.1371/journal.pone.0251768 (PMC8121324; doi:10.1371/journal.pone.0251768)

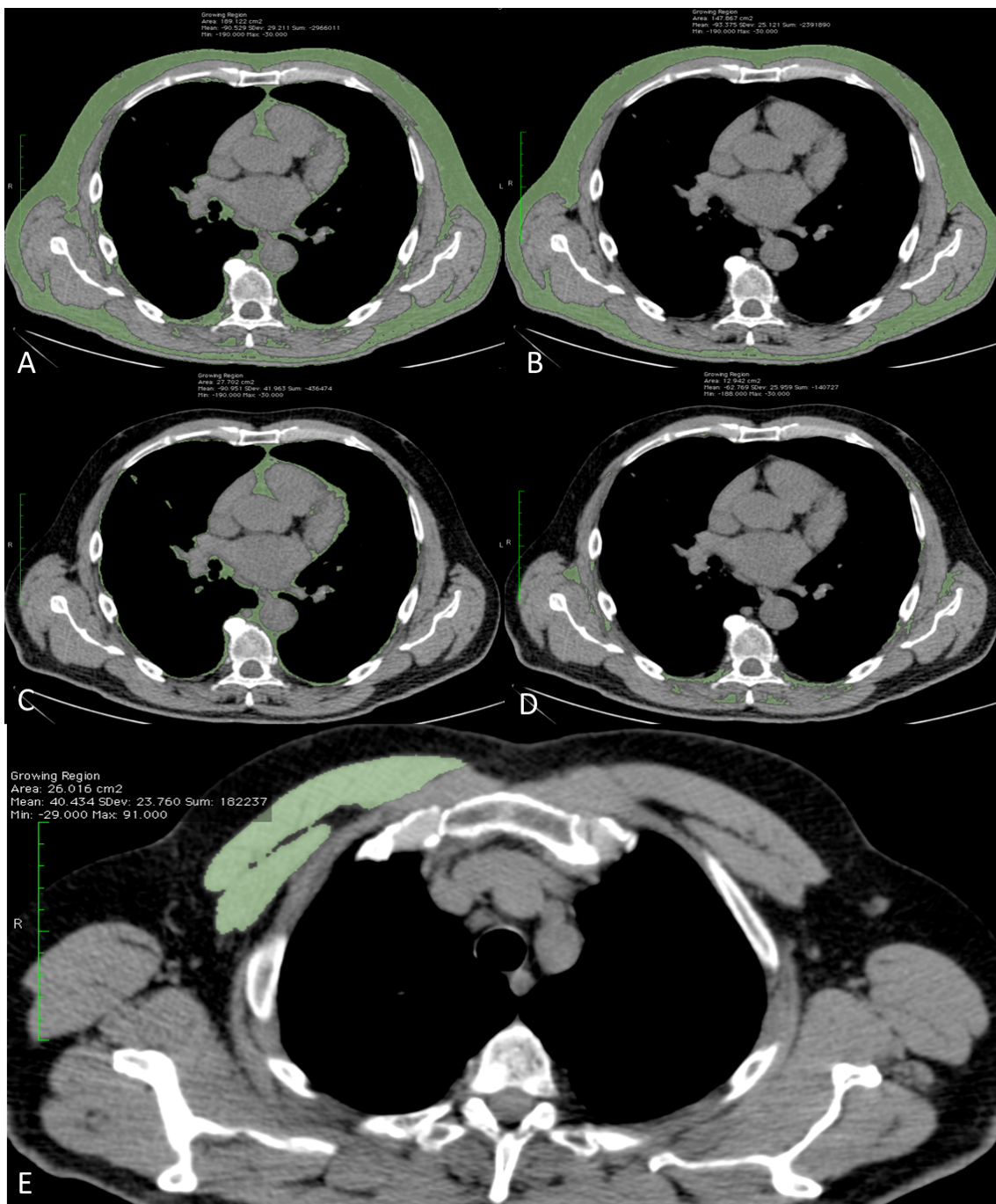

Supplement: S1 Fig — Total adipose tissue, TAT (A), subcutaneous adipose tissue, SAT (B), visceral adipose tissue, VAT (C), intermuscular adipose tissue, IMAT (D), were all measured at the level of T7-T8 vertebrae. Pectoral muscle area and density were measured on the right side at a level immediately superior to the aortic arch (E). (PDF) [file pone.0251768.s005.pdf]

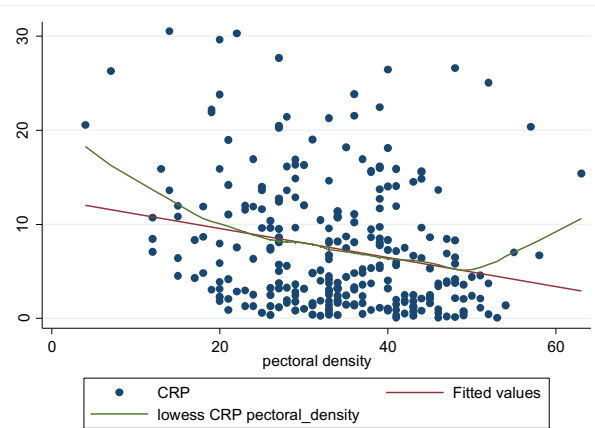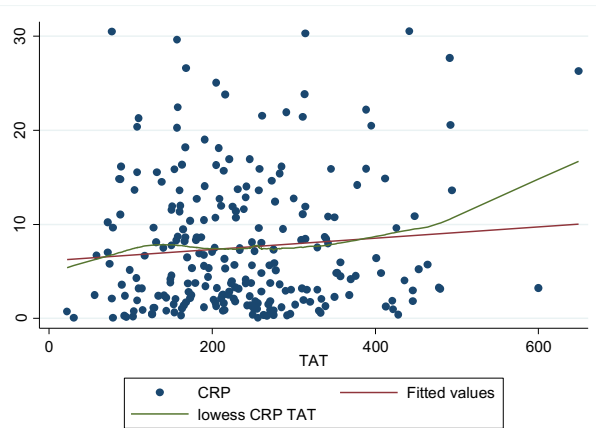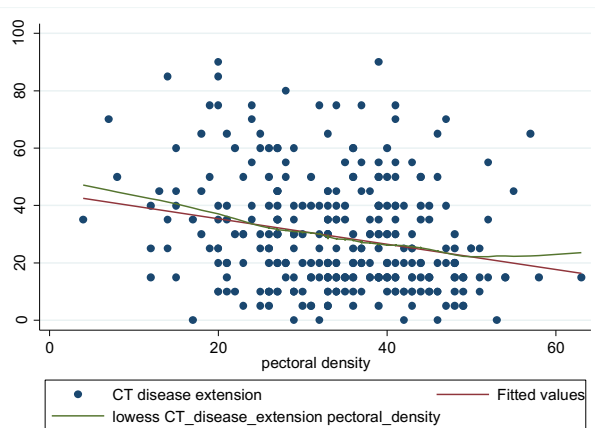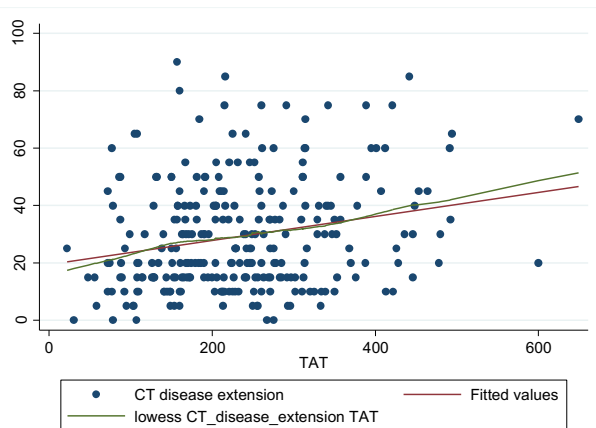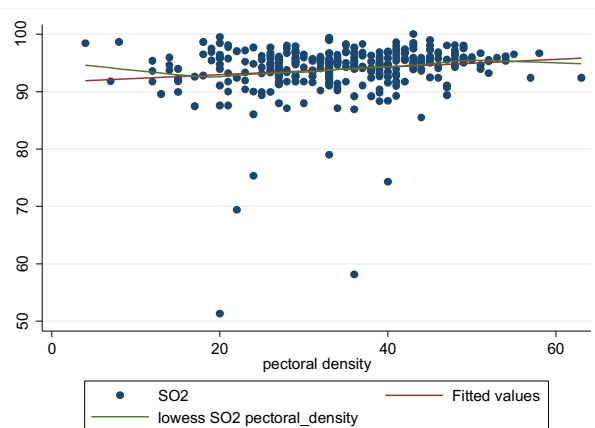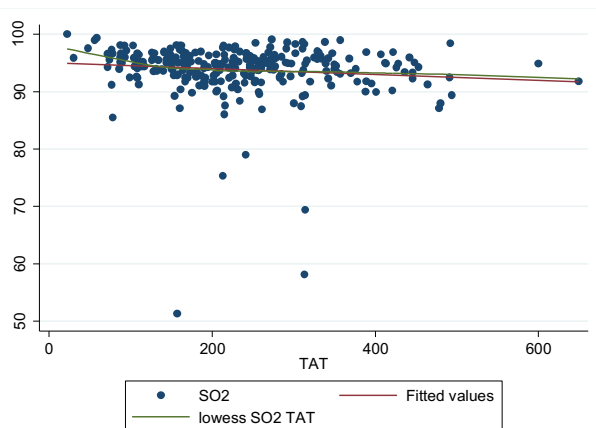

Supplement: S2 Fig — CRP: C-reactive protein; CT: Computed Tomography; SO2: oxygen saturation level; TAT: total adipose tissue area. (PDF) [file pone.0251768.s006.pdf]

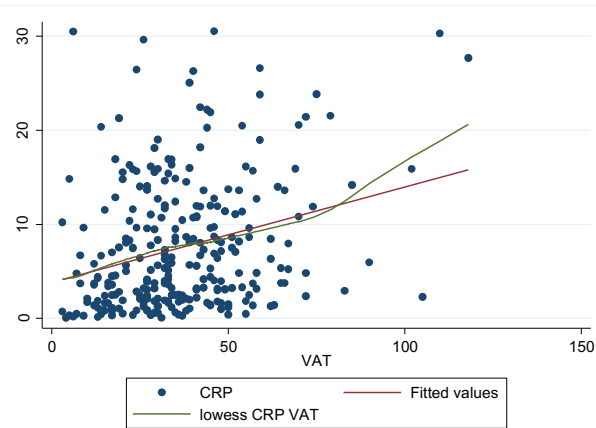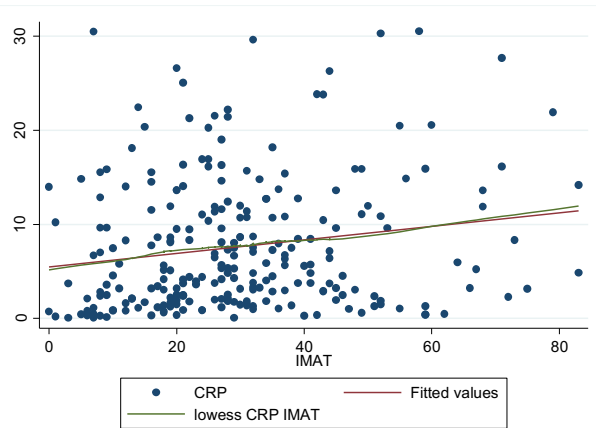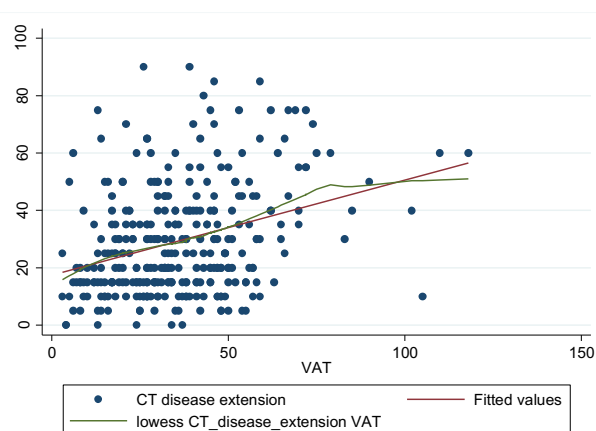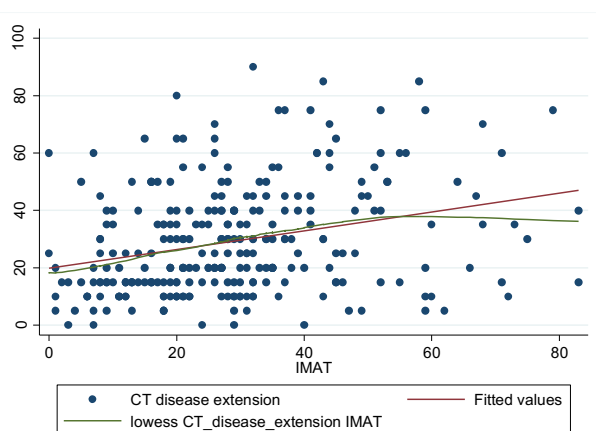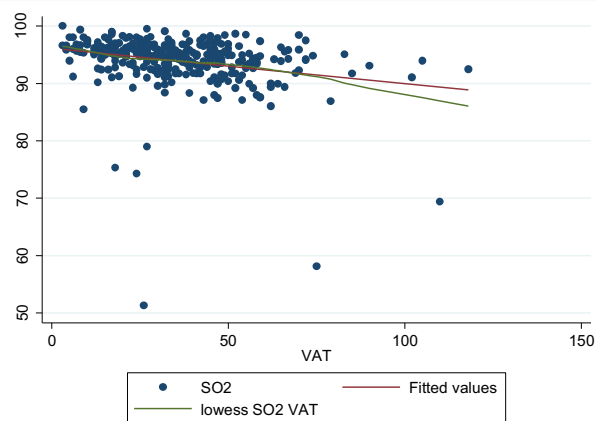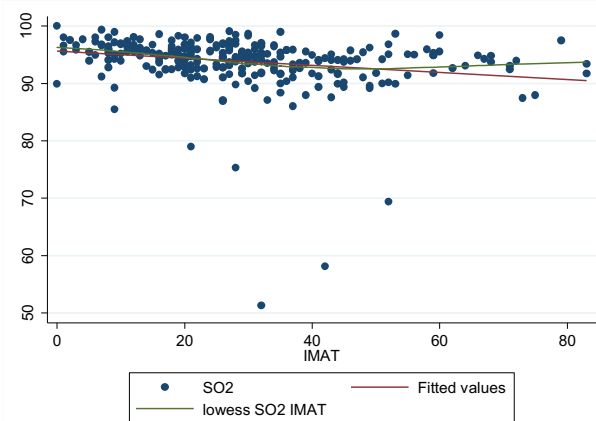

Supplement: S3 Fig — CRP: C-reactive protein; CT: Computed Tomography; IMAT: intermuscular adipose tissue area SO2: oxygen saturation level; VAT: visceral adipose tissue area. (PDF) [file pone.0251768.s007.pdf]
