# Supplementary material for: The impact of chest CT body composition parameters on clinical outcomes in COVID-19 patients
Source: PLoS One. 2021 May 14;16(5):e0251768. doi: 10.1371/journal.pone.0251768 (PMC8121324; doi:10.1371/journal.pone.0251768)
Supplement: Attachment — Submitted filename: Response to Reviewers.docx [file pone.0251768.s008.docx]

PONE-D-21-05721
The impact of chest CT body composition parameters on clinical outcomes in COVID-19 patients
PLOS ONE

Dear Dr. Massimo,

Thank you for submitting your manuscript to PLOS ONE. After careful consideration, we feel that it has merit but does not fully meet PLOS ONE’s publication criteria as it currently stands. Therefore, we invite you to submit a revised version of the manuscript that addresses the points raised during the review process.

Please submit your revised manuscript by 23 April. If you will need more time than this to complete your revisions, please reply to this message or contact the journal office at [plosone@plos.org](mailto:plosone@plos.org). When you're ready to submit your revision, log on to <https://www.editorialmanager.com/pone/> and select the 'Submissions Needing Revision' folder to locate your manuscript file.

Please include the following items when submitting your revised manuscript:

- A rebuttal letter that responds to each point raised by the academic editor and reviewer(s). You should upload this letter as a separate file labeled 'Response to Reviewers'.
- A marked-up copy of your manuscript that highlights changes made to the original version. You should upload this as a separate file labeled 'Revised Manuscript with Track Changes'.
- An unmarked version of your revised paper without tracked changes. You should upload this as a separate file labeled 'Manuscript'.

If you would like to make changes to your financial disclosure, please include your updated statement in your cover letter. Guidelines for resubmitting your figure files are available below the reviewer comments at the end of this letter.

If applicable, we recommend that you deposit your laboratory protocols in [protocols.io](http://protocols.io/) to enhance the reproducibility of your results. [Protocols.io](http://protocols.io/) assigns your protocol its own identifier (DOI) so that it can be cited independently in the future. For instructions see: [http://journals.plos.org/plosone/s/submission-guidelines#loc-laboratory-protocols](https://www.google.com/url?q=http://journals.plos.org/plosone/s/submission-guidelines%23loc-laboratory-protocols&source=gmail-imap&ust=1618904101000000&usg=AOvVaw1VHff9L6thhjFJx1s6Nn8z). Additionally, PLOS ONE offers an option for publishing peer-reviewed Lab Protocol articles, which describe protocols hosted on [protocols.io](http://protocols.io/). Read more information on sharing protocols at [https://plos.org/protocols?utm_medium=editorial-email&utm_source=authorletters&utm_campaign=protocols](https://www.google.com/url?q=https://plos.org/protocols?utm_medium%3Deditorial-email%26utm_source%3Dauthorletters%26utm_campaign%3Dprotocols&source=gmail-imap&ust=1618904101000000&usg=AOvVaw1pLDQUfYlvWjf8hx2AG5sk).

We look forward to receiving your revised manuscript.

Kind regards,

Francesco Di Gennaro
Academic Editor
PLOS ONE

Journal Requirements:

When submitting your revision, we need you to address these additional requirements.

1. Please ensure that your manuscript meets PLOS ONE's style requirements, including those for file naming. The PLOS ONE style templates can be found at

<https://journals.plos.org/plosone/s/file?id=wjVg/PLOSOne_formatting_sample_main_body.pdf> and

<https://journals.plos.org/plosone/s/file?id=ba62/PLOSOne_formatting_sample_title_authors_affiliations.pdf>

RE: these requirements have been checked.

2. Please provide additional details regarding participant consent. In the ethics statement in the Methods and online submission information, please ensure that you have specified what type you obtained (for instance, written or verbal, and if verbal, how it was documented and witnessed). If your study included minors, state whether you obtained consent from parents or guardians. If the need for consent was waived by the ethics committee, please include this information.

RE: the consent was verbal, when collected. Given the retrospective nature of the study, it was not always collected. A sentence has been added to better explain these points.

“Given the retrospective nature of the study, the Ethics Committee authorizes the use of a patient’s data without his/ her written informed consent if all reasonable efforts have been made to contact that patient.”

If needed, we can upload a copy of the written consent and a copy of the declaration which substitutes the informed consent in specific cases (death or impossibility to contact the patient).

3. We note that you have indicated that data from this study are available upon request. PLOS only allows data to be available upon request if there are legal or ethical restrictions on sharing data publicly. For information on unacceptable data access restrictions, please see <http://journals.plos.org/plosone/s/data-availability#loc-unacceptable-data-access-restrictions>.

In your revised cover letter, please address the following prompts:

3a) If there are ethical or legal restrictions on sharing a de-identified data set, please explain them in detail (e.g., data contain potentially identifying or sensitive patient information) and who has imposed them (e.g., an ethics committee). Please also provide contact information for a data access committee, ethics committee, or other institutional body to which data requests may be sent.

3b) If there are no restrictions, please upload the minimal anonymized data set necessary to replicate your study findings as either Supporting Information files or to a stable, public repository and provide us with the relevant URLs, DOIs, or accession numbers. Please see <http://www.bmj.com/content/340/bmj.c181.long> for guidelines on how to de-identify and prepare clinical data for publication. For a list of acceptable repositories, please see <http://journals.plos.org/plosone/s/data-availability#loc-recommended-repositories>.

RE: According to Italian law, anonymized data can only be made publicly available if there is potential for the re-identification of individuals (https://www.garanteprivacy.it). Furthermore, property of the data remains of the patient, who gave consent to use data for the objective of the study. Thus, the data underlying this study are available on request to researchers who intend to conduct research in the respect of confidentiality (even if anonymous data are provided, they should be published in aggregated form) and for studies with objectives consistent with those of the original study. In order to obtain data, approval must be obtained from the Area Vasta Emilia Nord (AVEN) Ethics Committee, who would check the consistency of the objective and planned analyses and would then authorize us to provide aggregated or anonymized data. Data access requests should be addressed to the Ethics Committee at CEReggioemilia@ausl.re.it as well as to the authors at the Epidemiology unit of AUSL - IRCCS of Reggio Emilia at info.epi@ausl.re.it, who are the data guardians.

The lead author (PGR) affirms that the manuscript is an honest, accurate, and transparent account of the study being reported; that no important aspect of the study has been omitted; and that any discrepancies from the study as planned have been explained.

4. We will update your Data Availability statement on your behalf to reflect the information you provide.

Thank you for stating the following in the Acknowledgments Section of your manuscript:

We thank Jacqueline Costa for the English language editing. This study is part of a larger

project supported by Ministry of Health (Grant number COVID-2020-12371808).

The following are the members of the Reggio Emilia COVID-19 Working Group: Massimo

Costantini, Roberto Grilli, Massimiliano Marino, Giulio Formoso, Debora Formisano, Paolo

Giorgi Rossi, Emanuela Bedeschi, Cinzia Perilli, Elisabetta La Rosa, Eufemia Bisaccia, Ivano

Venturi, Massimo Vicentini, Cinzia Campari, Francesco Gioia, Serena Broccoli, Marta Ottone,

Pierpaolo Pattacini, Giulia Besutti, Valentina Iotti, Lucia Spaggiari, Pamela Mancuso, Andrea

Nitrosi, Marco Foracchia, Rossana Colla, Alessandro Zerbini, Marco Massari, Anna Maria

Ferrari, Mirco Pinotti, Nicola Facciolongo, Ivana Lattuada, Laura Trabucco, Stefano De Pietri,

Giorgio Francesco Danelli, Laura Albertazzi, Enrica Bellesia, Simone Canovi, Mattia

Corradini, Tommaso Fasano, Elena Magnani, Annalisa Pilia, Alessandra Polese, Silvia Storchi

Incerti, Piera Zaldini, Efrem Bonelli, Bonanno Orsola, Matteo Revelli, Carlo Salvarani,

Carmine Pinto, Francesco Venturelli, Elisabetta Teopompi, Annalisa Gallina, Annalisa

Bertellini, Stefania Costi, Stefania Fugazzaro.

We note that you have provided funding information that is not currently declared in your Funding Statement. However, funding information should not appear in the Acknowledgments section or other areas of your manuscript. We will only publish funding information present in the Funding Statement section of the online submission form.

Please remove any funding-related text from the manuscript and let us know how you would like to update your Funding Statement. Currently, your Funding Statement reads as follows:

The authors received no specific funding for this work.

Please include your amended statements within your cover letter; we will change the online submission form on your behalf.

RE: Data collection started in 2020 without any funding source. However, final analyses (and future publication) have been partially funded by the Italian Ministry of Health, Grant number COVID-2020-12371808. For this reason, we wrote “This study is part of a larger project supported by Ministry of Health (Grant number COVID-2020-12371808).” in the acknowledgment section. In the new version we have removed this sentence from the acknowledgment section but we should probably change the Funding Statement explaining this partial funding.

Please review your reference list to ensure that it is complete and correct. If you have cited papers that have been retracted, please include the rationale for doing so in the manuscript text, or remove these references and replace them with relevant current references. Any changes to the reference list should be mentioned in the rebuttal letter that accompanies your revised manuscript. If you need to cite a retracted article, indicate the article’s retracted status in the References list and also include a citation and full reference for the retraction notice.

RE: we have added an erratum in ref number 2.

Additional Editor Comments:

dear authors follow reviewer suggestions to improve your paper


[Note: HTML markup is below. Please do not edit.]

Reviewers' comments:

Reviewer's Responses to Questions

**Comments to the Author**

1. Is the manuscript technically sound, and do the data support the conclusions?

The manuscript must describe a technically sound piece of scientific research with data that supports the conclusions. Experiments must have been conducted rigorously, with appropriate controls, replication, and sample sizes. The conclusions must be drawn appropriately based on the data presented.

Reviewer #1: Yes

Reviewer #2: Yes

Reviewer #3: Yes

2. Has the statistical analysis been performed appropriately and rigorously?

Reviewer #1: Yes

Reviewer #2: Yes

Reviewer #3: Yes

3. Have the authors made all data underlying the findings in their manuscript fully available?

The [PLOS Data policy](https://www.google.com/url?q=http://www.plosone.org/static/policies.action%23sharing&source=gmail-imap&ust=1618904101000000&usg=AOvVaw2-_LUYNu7_ShTc2iflWCkh) requires authors to make all data underlying the findings described in their manuscript fully available without restriction, with rare exception (please refer to the Data Availability Statement in the manuscript PDF file). The data should be provided as part of the manuscript or its supporting information, or deposited to a public repository. For example, in addition to summary statistics, the data points behind means, medians and variance measures should be available. If there are restrictions on publicly sharing data—e.g. participant privacy or use of data from a third party—those must be specified.

Reviewer #1: Yes

Reviewer #2: Yes

Reviewer #3: Yes

4. Is the manuscript presented in an intelligible fashion and written in standard English?

PLOS ONE does not copyedit accepted manuscripts, so the language in submitted articles must be clear, correct, and unambiguous. Any typographical or grammatical errors should be corrected at revision, so please note any specific errors here.

Reviewer #1: Yes

Reviewer #2: Yes

Reviewer #3: Yes

5. Review Comments to the Author

Please use the space provided to explain your answers to the questions above. You may also include additional comments for the author, including concerns about dual publication, research ethics, or publication ethics. (Please upload your review as an attachment if it exceeds 20,000 characters)

Reviewer #1: The manuscript was good written and discussed.
It can be accepted.
Introduction: was good written
Materials and Methods: were appropriately written and explained briefly
Results: were good written
Discussion: was good written

Reviewer #2: Congratulations

Reviewer #3: This is a relevant study and performed in a correct way scientifically. It addresses relevant research questions and analyses these in an appropriate way. The weaknesses are that it is retrospective and interpolates abdominal body composition parameters from CT slices of the chest. All data are available in the article and the supplementary tables and figures. There are some information lacking in the legends, e.g. what information is given within the brackets in Table S1 and what test i applied in Table S2?

RE: We thank the Reviewers for the overall positive judgement of our work. We also thank the Reviewers for the suggestion to improve the manuscript.

In the new version we have added a sentence to the limitation section: “First, data collection was retrospective and chest CT body composition parameters are less validated than those obtained from abdominal CT at L3 level [18, 27-28].”

We have also added the lacking information to the legends in S1 “Spearman's rank correlation coefficients are reported with respective p-values between brackets.” and S2 “… by an unadjusted logit regression model. β coefficients with respective 95% confidence intervals are reported.”

6. PLOS authors have the option to publish the peer review history of their article ([what does this mean?](https://www.google.com/url?q=https://journals.plos.org/plosone/s/editorial-and-peer-review-process%23loc-peer-review-history&source=gmail-imap&ust=1618904101000000&usg=AOvVaw0lrGtCKF8sX55gGNikwybF)). If published, this will include your full peer review and any attached files.


If you choose “no”, your identity will remain anonymous but your review may still be made public.


**Do you want your identity to be public for this peer review?** For information about this choice, including consent withdrawal, please see our [Privacy Policy](https://www.google.com/url?q=https://www.plos.org/privacy-policy&source=gmail-imap&ust=1618904101000000&usg=AOvVaw3mVvF0sEnHwjn7CuibWkt8).

Reviewer #1: **Yes:** Abd El Raouf, Mustafa

Reviewer #2: No

Reviewer #3: No

[NOTE: If reviewer comments were submitted as an attachment file, they will be attached to this email and accessible via the submission site. Please log into your account, locate the manuscript record, and check for the action link "View Attachments". If this link does not appear, there are no attachment files.]

While revising your submission, please upload your figure files to the Preflight Analysis and Conversion Engine (PACE) digital diagnostic tool, [https://pacev2.apexcovantage.com/](https://www.google.com/url?q=https://pacev2.apexcovantage.com/&source=gmail-imap&ust=1618904101000000&usg=AOvVaw0ZtXLPopgf64iIBmybb_Dk). PACE helps ensure that figures meet PLOS requirements. To use PACE, you must first register as a user. Registration is free. Then, login and navigate to the UPLOAD tab, where you will find detailed instructions on how to use the tool. If you encounter any issues or have any questions when using PACE, please email PLOS at [figures@plos.org](mailto:figures@plos.org). Please note that Supporting Information files do not need this step.
